# Uncertainty quantification of reference-based cellular deconvolution algorithms

**DOI:** 10.1080/15592294.2022.2137659

**Published:** 2022-12-20

**Authors:** Dorothea Seiler Vellame, Gemma Shireby, Ailsa MacCalman, Emma L Dempster, Joe Burrage, Tyler Gorrie-Stone, Leonard S Schalkwyk, Jonathan Mill, Eilis Hannon

**Affiliations:** a University of Exeter Medical School, University of Exeter, Exeter EX2 5DW, UK; b School of Biological Sciences, University of Essex, Colchester CO4 3SQ, UK

**Keywords:** DNA methylation, epigenetic epidemiology, illumina 450K array, Illumina EPIC array, cellular heterogeneity

## Abstract

The majority of epigenetic epidemiology studies to date have generated genome-wide profiles from bulk tissues (e.g., whole blood) however these are vulnerable to confounding from variation in cellular composition. Proxies for cellular composition can be mathematically derived from the bulk tissue profiles using a deconvolution algorithm; however, there is no method to assess the validity of these estimates for a dataset where the true cellular proportions are unknown. In this study, we describe, validate and characterize a sample level accuracy metric for derived cellular heterogeneity variables. The CETYGO score captures the deviation between a sample’s DNA methylation profile and its expected profile given the estimated cellular proportions and cell type reference profiles. We demonstrate that the CETYGO score consistently distinguishes inaccurate and incomplete deconvolutions when applied to reconstructed whole blood profiles. By applying our novel metric to >6,300 empirical whole blood profiles, we find that estimating accurate cellular composition is influenced by both technical and biological variation. In particular, we show that when using a common reference panel for whole blood, less accurate estimates are generated for females, neonates, older individuals and smokers. Our results highlight the utility of a metric to assess the accuracy of cellular deconvolution, and describe how it can enhance studies of DNA methylation that are reliant on statistical proxies for cellular heterogeneity. To facilitate incorporating our methodology into existing pipelines, we have made it freely available as an R package (https://github.com/ds420/CETYGO).

## Introduction

Due to the dynamic nature of the epigenome and its plasticity in response to environmental exposures [1–4], there is increasing interest in the role it plays in the aetiology of disease [5]. However, this very facet of the epigenome makes epigenetic epidemiology studies inherently more complex to design and liable to confounding compared to studies of DNA sequence variation [6,7]. One major difference is that an individual’s genetic sequence is identical in all cells, and therefore it does not matter from which tissue DNA is isolated prior to genotyping. In contrast, the epigenome orchestrates gene expression changes that underpin cellular differentiation; consequently, cell types can be defined by their epigenetic profiles [8]. It has previously been shown that variation between cell types is greater than inter-individual variation within a cell type [9,10].

The majority of studies to date have focused on a single epigenetic modification, DNA methylation (DNAm), and generated genome-wide profiles from bulk tissues (e.g., whole blood) using high throughput microarrays [11]. A critical challenge in these studies is that bulk tissue is a heterogeneous mix of different cell types. The epigenetic profile of a bulk tissue is the average across the profiles of the constituent cell types. If the composition of these cell types, specifically the proportions of each cell type, varies across the population under study, and varies in a manner that correlates with the outcome of interest, this will lead to false positive associations at sites in the genome that differ between cell types [12,13]. As a result, epigenome-wide association analyses routinely include quantitative covariates that capture the heterogeneity in cellular composition across a dataset. As experimentally derived cell counts are often unavailable, proxies for cellular composition can be derived from the bulk tissue profile using a deconvolution algorithm. The goal of these statistical methodologies is to generate a series of continuous variables that reflect the underlying cellular heterogeneity of each sample. Deconvolution algorithms can be separated into two classes. Firstly, supervised methods that incorporate reference profiles for relevant cell types – generated from purified cell populations – and estimate proportions for this specified set of cell types (known as reference-based algorithms) [14–20]. Secondly, those that do not use any reference data and generate an unlimited set of variables that are not directly attributed to any particular cell type (known as reference-free algorithms) [21–24].

In tissues for which reference profiles are available, reference-based deconvolution algorithms are most commonly used, likely due to the ease of interpretation. Specifically the constrained projection methodology proposed by Houseman, often referred to as ‘Houseman’s method,’ is normally used. There have been a number of studies that have aimed to validate the application of these methods by testing their performance against experimentally or computationally derived ‘bulk’ profiles of fixed cellular compositions [18,25,26]. These have primarily focused on the prediction of the major blood cell types from whole blood. Typically, accuracy is reported at the group level, i.e., a single correlation or error statistic across a number of samples, which is then assumed to be representative for all future applications. In prediction modelling, great attention is paid to ensuring that the training data is representative of the testing data so that the predictions are valid. The vast majority of whole blood epigenetic studies use the same reference dataset generated from six adult males [27] to determine cellular composition, regardless of the age, sex, ethnicity, or disease status characteristics, with little consideration given to whether it is representative of the cohort being tested. Mathematically, there is nothing to prevent a deconvolution algorithm, based on any reference panel of cell types, from being applied to a profile generated from any bulk tissue. As an extreme example, we could input data derived from brain tissue to a model that outputs estimates of the composition of blood cell types and obtain values, due to the mathematical constraints that are plausible (i.e., between 0 and 1). In a less extreme example, it is unknown how important demographic features (e.g., age, sex, or ethnicity) of the samples in the reference panel affect prediction in samples characterized by different demographics. Currently, there is no method to assess the validity of cellular composition estimates for a single sample, or indeed, a dataset where the true cellular proportions are unknown. If the quality of the deconvolution varies either, across studies or within a study, then the utility of these variables as confounders needs to be reconsidered. This could be especially problematic if the accuracy of the deconvolution is systematically biased and is related to any other confounders such as age or sex. Understanding how reliable a set of cellular heterogeneity variables are for any individual sample is of increasing importance, as the interest in quantifying cellular composition has moved beyond just adjusting for it in epigenome-wide association studies, with these estimates also being analysed as variables of interest in their own right [28, 29, 30].

In this study, we propose an accuracy metric that quantifies the **CE**ll **TY**pe deconvolution **GO**odness (CETYGO) score of a set of cellular heterogeneity variables derived from a genome-wide DNAm profile for an individual sample. While our method is applicable to any reference-based deconvolution algorithm, and any reference panel of cell types, to demonstrate the utility of our approach we limit our characterization to the Houseman algorithm and two common panels of blood cell types, which represents the majority of applications. We demonstrate that CETYGO indexes the accuracy of the prediction of cellular composition with simulations in which we manipulated the performance of the deconvolution. We then profile the statistical properties of the CETYGO score by applying it to a number of empirical datasets, to provide guidance on how it can be incorporated into whole blood DNAm studies. Finally, we use the CETYGO score to determine if they are any biases in the effectiveness of existing blood cell type reference panels. To enable the wider research community to incorporate our proposed error metric into their analyses, we have provided our methodology in an R package, CETYGO, as well as adding functions to the wateRmelon package [31].

## Materials and methods

### Mathematical derivation of the CETYGO score

The DNAm profile of a bulk tissue can be defined as the sum of DNAm levels measured in the constituent cell types weighted by the proportion of total cells represented by that cell type. Mathematically we can represent this as

(1) $$B_{i,j}=\overset{N}{\underset{k=1}{\sum }}p_{i,k}C_{i,j,k}$$

where

- *B_i,j_* represents the DNAm level in the bulk tissue for sample i at site j
- *p_i,k_* represents the proportion of cell type k in sample i
- *C_i,j,k_* represents the DNAm level for sample i at site j in cell type k, for N different cell types.

Typically in an epidemiological study, only the bulk tissue DNAm profile (*B_i,j_*) is measured. However, as cellular composition is an important confounder, it is desirable to know or estimate *p_i,k_* for all (major) cell types. Methods for this purpose, such as Houseman’s constraint projection approach, have been proposed that take advantage of reference profiles (i.e., *C_i,j,k_*) available to the research community to enable them solve for the unknown *p_i,k_*. This is achieved by selecting *M* DNAm sites that are highly discriminative of the cell types we want to estimate the proportions of. By definition, these sites exhibit low variation across individuals, and therefore it does not theoretically matter that we have not measured them in the same samples that we have bulk profiles from. If the estimated cell proportions (denoted $\hat{p_{i,k}}$) are accurate then the expected bulk tissue profile given this composition of cell types should closely resemble the observed data. We can substitute our estimated cell proportions, $\hat{p_{i,k}}$, back into Equation 1, to calculate the expected profile of DNAm values (Equation 2) using the reference data to provide values for the cell-specific DNAm levels.

(2) $$\hat{B_{i,j}}=\overset{N}{\underset{k=1}{\sum }}\hat{p_{i,k}}C_{i,j,k}$$

We define our error metric, CETYGO, as the root mean square error (RMSE) between the observed bulk DNAm profile and the expected profile across the *M* cell type specific DNAm sites used to perform the deconvolution, calculated from the estimated proportions for the *N* cell types (Equation 3). By definition, 0 is the lowest value the CETYGO score can take and would indicate a perfect estimate. Higher values of the CETGYO score are indicative of larger errors and therefore a less accurate estimation of cellular composition.

(3) $$CETYGO_{i}=RMSE\left(B_{i},\hat{B_{i}}\right)=\sqrt{\frac{\sum_{1}^{M}\left({\left(B_{i,j}-\hat{B_{i,j}}\right)}^{2}\right)}{M}}$$

### Purified blood cell type reference panels

Genome-wide DNAm profiles for purified blood cell types generated using the Illumina 450 K and EPIC microarray were obtained via the *FlowSorted.Blood.450k* and *FlowSorted.Blood.EPIC* R packages and formatted into matrices of beta values using commands from the *minfi* [32] R package. From the 450 K reference panel, we selected the six blood cell types that are mostly commonly used (B-cells, CD4+ T-cells, CD8+ T-cells, granulocytes, monocytes and natural killer cells) which were purified from whole blood from 6 Swedish male individuals using flow cytometry [27]. The mean purity of these samples was 92% (range 72–99%). The EPIC reference panel contains profiles from antibody bead sorted neutrophils (n = 6), B-cells (n = 6), monocytes (n = 6), natural killer cells (n = 6), CD4+ T-cells (n = 7), and CD8+ T-cells (n = 6) [26] from male and female donors from a broad range of ethnicities (African-American, East-Asian, Indo-European, multiple/admixed). The average purity of these samples was 95% (range 88%-99%). Prior to training any deconvolution models, both reference datasets were filtered to only include autosomal DNAm sites.

### Generation of deconvolution models and simulated whole blood profiles

To test the performance of CETYGO against a known truth, we trained a series of Houseman constraint projection deconvolution models using reference data for different combinations of purified blood cell types (**Supplementary Figure 1**). These were then tested against reconstructed whole blood DNAm profiles where we combined cell-specific profiles in a weighted linear sum of pre-specified proportions of each cell type. Note that when we refer to different models, these differ by way of the cell types included in the reference panel and the datasets from which the samples were taken rather than different algorithms. Depending on the specific testing framework, the training data comprised of all available samples that matched the relevant criteria and were not selected to be part of the testing data, such that the train and test data consisted of distinct sets of samples. It should be noted though that in some scenarios they were from the sample experimental batch, and plausibly share technical, batch-specific effects. We modified the *minfi* approach for implementing Houseman’s constrained projection methodology to omit the step within *estimateCellCounts()* where the training and test data are normalized together, in order to explore the effect of normalization. This adaptation means that the cellular deconvolution and CETYGO calculation can be applied directly to a matrix of beta values, rather than requiring the raw data stored in an RGSet object. This makes it straightforward and computationally efficient to apply new reference panel (or include a new error metric) to an existing dataset. After selecting the training data, the deconvolution model was formulated as follows. An ANOVA was performed across all samples in the training data to identify sites that are significantly different (p value < 1 × 10^−8^) between the blood cell types, selecting 100 sites per cell type (50 hypermethylated and 50 hypomethylated). These sites are then used to solve Equation 1 using quadratic programming, in essence a least squares minimization, with the constraint that the proportions are greater than or equal to 0.

In the first simulation analysis, we had six different combinations of training and testing data using the two reference panels. Within each reference panel (450 K and EPIC), across reference panels without normalization (450 K to EPIC and EPIC to 450 K) and across reference panels after stratified quantile normalization as implemented in *minfi* of the combined training and test dataset (450 K to EPIC and EPIC to 450 K). To construct whole blood profiles for testing we isolated one sample of each cell type. When testing samples were selected from the 450 K reference data, we selected a single individual as the test case and took all their purified samples, with all the samples from the other five individuals used for the training data. This meant there were a maximum of 6 testing iterations (as there are 6 individuals). When testing samples were selected from the EPIC reference data, we randomly selected a test sample for each cell type (as they do not come from the same set of individuals), and repeated this process 10 times to get multiple sets of test data. We constructed whole blood profiles (i.e., the test data) as a linear sum of these cell-specific profiles in a fixed ratio and a defined proportion of noise. Specifically the test profiles where generated using the equation,

(4) $$B_{j}=\overset{N}{\underset{k=1}{\sum }}p_{k}C_{j,k}+\rho \varepsilon_{j}$$

Where

*B_j_* represents the simulated DNAm level in the bulk tissue at site j.

*p_k_* represents the proportion of cell type k which were standardized for these series of simulations to the mean proportions reported in Reinius et al. [27] (**Supplementary Table 1**).

*C_j,k_* represents the DNAm level from the test sample for in cell type k at site j.

ρ is the proportion of ‘noise’ and took the values 0, 0.01, 0.02, …, 0.1, 0.12, 0.14, … 0.5.

ε_j_ is a random variable taken from a uniform distribution bounded by 0 and 1.

In total 31 simulated ‘noisy’ blood profiles were tested for each iteration of each deconvolution model.

In the second simulation analysis, we focused on a single reference panel, the 450 K reference panel. Here we tested a series of deconvolution models, where each cell type was omitted in turn from the reference panel, prior to training the model. Each of these leave one out models, was then tested against simulated whole blood profiles constructed from all six cell types. The five cell types included in the training data were combined for the test data in fixed ratios calculated from the mean proportions reported by Reinius et al. (**Supplementary Table 1**), with the omitted cell type included at increasing proportions (0.1, 0.2, …, 0.9). We used the same process to select testing samples as described before, meaning that each of the leave one out models was tested against 9 simulated whole blood profiles in 6 different train test permutations.

In the third simulation analysis, we again focused on a single reference panel, the 450 K reference panel. Here, we tested all possible deconvolution models, containing between 3 and 5 of the 6 blood cell types, a total of 41 combinations. This time we tested the full spectrum of whole blood profiles in 0.1 units, where each cell type represented at least 0.1, up to a maximum of 0.5. In total 126 possible profiles were generated, where every combination of blood cell types was considered.

### Profiling the performance of CETYGO in empirical datasets

A summary of the 17 datasets used to profile CETYGO is provided in **Supplementary Table 2**. Datasets 2–9, 14, and 15 were generated by our group at the University of Exeter (www.epigenomicslab.com) and have been previously published. The pre-processing and normalization of these datasets is as described in the corresponding manuscripts. Datasets 1 and 16 were also generated by our group and are currently unpublished. They followed a standard QC pipeline and were normalized using *dasen()* in the *wateRmelon* package [31]. Datasets 10–13 and 17 are publically available datasets obtained from GEO (https://www.ncbi.nlm.nih.gov/geo/). These data were put through a quality control pipeline which included checking the quality of the DNAm data (signal intensity, bisulphite conversion and detection p-values) prior to normalization using *dasen()* in the *wateRmelon* package [31]. For all datasets cellular deconvolution and the calculation of CETYGO was applied using a model trained with all samples for 6 cell types from the 450 K reference panel.

To characterize the relationship between data quality metrics and CETYGO, we used an expanded version of Dataset 3 which retained the samples that failed quality control for either a technical or biological reason (n = 725). For this data we imported the raw signal intensities from the idat files for all samples using the *wateRmelon* package [31]. Signal intensities for each sample were summarized as the median methylated (M) and unmethylated (U) intensity across all sites. Bisulfite conversion efficiency was calculated as the median beta value across 10 fully methylated control probes and converted to a percentage. Samples were then processed through *pfilter()* using the default settings. A sample was classed as a technical failure if either median signal intensity metric was less than 500, the bisulfite conversion statistic was less than 80% or it failed *pfilter*(). In total 62 samples were classed as technical failures. Note these thresholds may not match up with the thresholds implemented in the quality control pipeline described in the original manuscript. All 725 samples were then normalized using *dasen* and cellular deconvolution and their CETYGO score estimated.

In order to test the effect of normalizing the reference panel DNAm dataset (i.e., training data) with the bulk tissue dataset (i.e., the test data) we imported the raw signal intensities for Dataset 1. We the re-normalized these data in conjunction with the reference panel prior to performing cellular deconvolution and the calculation of CETYGO. To facilitate this we have adapted the *estimateCellCounts*() function in *minfi* [32] to a new function *estimateCellCountsWithError*() which additionally calculates CETYGO alongside performing the reference-based deconvolution. We made no other edits to the function and as such the data pre-processing is unchanged from the original function. This means that in these analyses the sex chromosomes were retained for both normalization and the selection of cell-specific sites for estimated cellular composition. These values of CETYGO were compared to CETYGO calculated as described above using the dasen normalized betas that were not normalized with the reference panel.

To compare the error associated with cellular deconvolution and the error associated with estimated age using an epigenetic clock, we implemented a robust regression model using the *rlm* package in R. A p-value for the co-efficient to test if it was non-zero was calculated using a Wald test as implemented in the *sfsmisc* R package.

### Ethical approval

The study was approved by the University of Exeter Medical School Research Ethics Committee (reference number 13/02/009).

### Data and code availability

The DNAm data used in this study are available as R packages or via GEO (see **Supplementary Table 2** for details). We have provided the code for calculating the CETYGO score as an R package available via GitHub (https://github.com/ds420/CETYGO). The code to reproduce the analyses in this manuscript using our R package are also available via GitHub (https://github.com/ejh243/CETYGOAnalyses).

## Results

### CETYGO indexes the accuracy of cellular composition estimates in whole blood

The objective of this study was to define, validate and characterize a novel metric that can be used to assess the accuracy of DNAm-based cellular deconvolution in an individual sample. The CETYGO score captures the deviation between the observed DNAm profile and the expected profile for the given set of estimated cell type proportions, where values close to 0 indicate accurate estimates of cellular composition.

**Figure 1.**
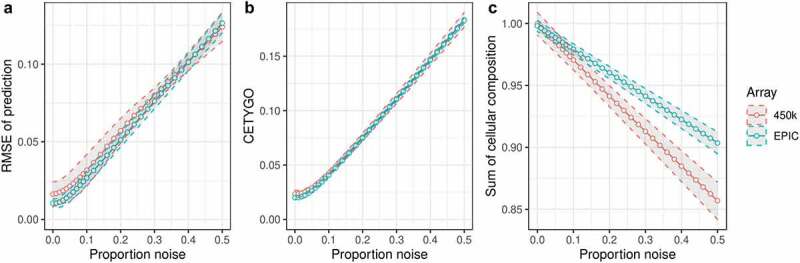
CETYGO captures variation in accuracy of cellular deconvolution in whole blood. Line graphs plotting the error associated with estimating the cellular proportions of reconstructed whole blood profiles with increasing proportion of noise (x-axis). Where the y-axis presents **A**) the root mean square error (RMSE) between the fixed cellular proportions used to construct the whole blood profiles and the estimated proportions generated with Houseman’s method, **B**) the error metric CETYGO and **C**) the sum of all proportions estimated. The points represent the mean value and the dashed lines the 95% confidence intervals calculated across multiple simulations. The two lines represent simulations constructed from reference data generated from two different platforms, the Illumina 450K and EPIC BeadChip microarrays.

In order to test whether our proposed error metric CETYGO successfully captures inaccurate cellular heterogeneity estimates, we manufactured a series of bulk whole blood profiles where the cellular composition was known and could be estimated with varying degrees of accuracy. This was achieved by standardizing the ratios of the constituent blood cell types and adding an increasing proportion of random ‘noise,’ which could reflect either biological variation, technical artefacts or imprecision in the assay. These simulations were run separately for a reference panel of blood cell types profiled with the 450K array [27] and one profiled with the EPIC array [26] (see **Materials and Methods**). The hypothesis is that as the proportion of noise increases, the estimation of cellular composition will be less accurate and the CETYGO score should correlate with the proportion of noise in the whole blood sample. To confirm that our simulation framework was fit for purpose, we calculated the RMSE between the fixed cell type proportions used to construct the whole blood profiles and the predicted values, observing that profiles with a higher proportion of noise were characterized by larger deviations from the truth (Figure 1(a)). Having manufactured a spectrum of inaccurate deconvolutions, we were able to determine whether the CETYGO score changed as a function of noise, finding that it successfully indexed accuracy with a monotonic relationship between the proportion of noise in a bulk sample and the CETYGO score (Figure 1(b)). We observed that for small proportions of noise (between 0 and 0.05) the accuracy estimates don’t vary very much, but once the proportion of noise goes above 0.05, the effect of additional noise on accuracy starts to accumulate. We also found that when the predictions were less accurate, the total sum of all estimated cell types for a sample was less than one and decreased as noise increased (Figure 1(c)).

In our simulation framework, we tested two independent reference datasets [26,27], generated using different versions of the Illumina BeadChip array and incorporating subtly different panels of cell types, either granulocytes or neutrophils, with the granulocytes fraction being 90% neutrophils. We subsequently repeated the simulation framework, but this time training the model using one reference panel (either 450K or EPIC) and testing it in simulations formulated from the other reference panel, limiting these analyses to the five cell types shared between the two reference panels. This would allow us to explore how batch and normalization strategy influences the accuracy of cellular deconvolution. These results showed the same general pattern across the different train-test pairings, where the CETYGO score captured decreasing accuracy in estimates of cellular composition (**Supplementary Figure 2**). Differences between datasets did lead to slightly increased imprecision at lower proportions of noise, but this scenario is arguably more representative of the typical application of cellular deconvolution algorithms, where the reference panel and bulk tissue test data are generated in different laboratories. Interestingly, we observed that when the training data was generated with the 450K array and applied to simulated bulk data generated from the EPIC array, the deconvolution was marginally more accurate potentially indicative of reduced signal-to-noise. This could be due to improved technical performance with the newer EPIC array or due to the fact that the reference samples had higher purity statistics. In general, whether the two batches of data were normalized together or not, there was no clear bias on deconvolution accuracy. When the EPIC array training data was used there was a minimal difference in deconvolution accuracy, measured by either RMSE (**Supplementary Figure 2A**), or the CETYGO score (**Supplementary Figure 2B**). Of interest, though, if the 450K training panel was used there was a moderate effect on RMSE with the direction of effect dependent on the proportion of noise. When noise is low (<0.1) normalizing the data together was associated with a smaller error, when noise was high (>0.1) normalizing the data separately was associated with a smaller error. This complex behaviour suggests technical characteristics of the reference panel itself (e.g., technology, data quality or cell purity) are more important than normalization strategy. Given the slightly more accurate performance, all subsequent analyses were performed with the 450K reference panel only.

### CETYGO is inflated when applied to incomplete cellular reference panels

**Figure 2.**
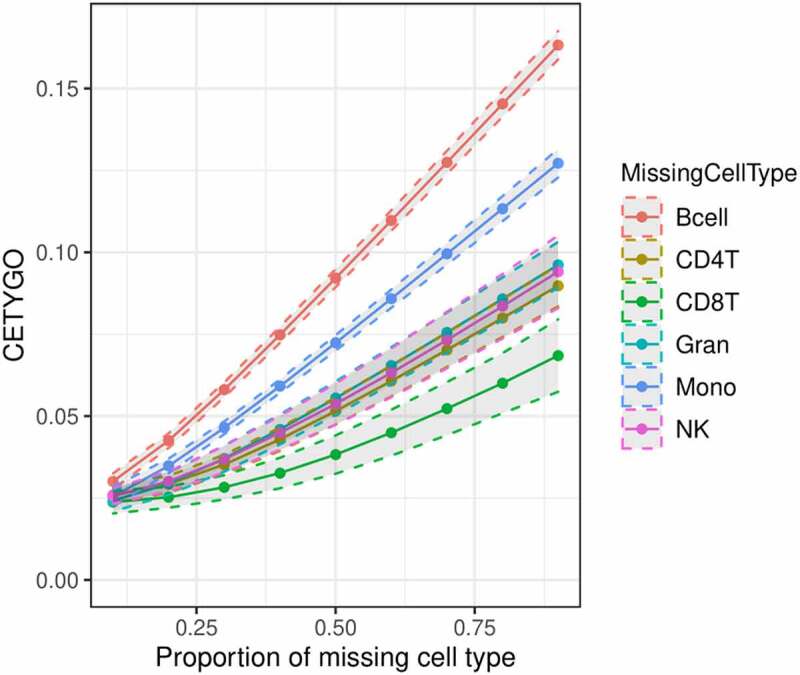
Cell type dependent effects on accuracy when omitted from reference based cellular deconvolution algorithms. Line graph of the error associated with estimating the cellular proportions of reconstructed whole blood profiles where the reference panel is missing one of six cell types. Each coloured line represents a different cell type being omitted from the reference panel, but included in the reconstructed whole blood profiles used for testing. Plotted is the proportion in the testing profile that the missing cell type is set to occupy (x-axis) against the error, measured using the CETYGO score, of the deconvolution (y-axis). The points represent the mean value and the dashed lines the 95% confidence intervals calculated across multiple simulations.

**Figure 3.**
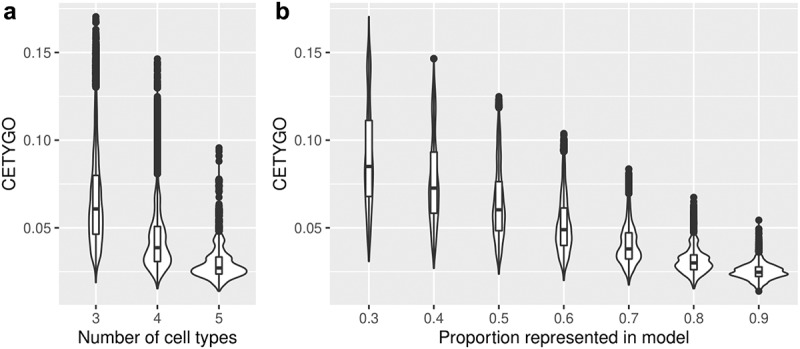
The accuracy of cellular heterogeneity estimation increases as the reference panel becomes more representative. Violin plots of the error associated with estimating the cellular proportions of reconstructed whole blood profiles where the reference panel is missing between one and three cell types. Each violin plot shows the distribution of the error, measured using CETYGO, of the deconvolution (y-axis) grouped by **A**) the number of cell types included in the reference panel and **B**) the proportion of cells in the reconstructed whole blood profile that are from cell types included in the reference panel.

Another scenario where inaccurate deconvolutions are likely to occur is when the reference panel of cell types for deconvolution is incomplete. When implementing Houseman’s method to solve for cellular composition proportions, there is an option to enforce a constraint such that the sum of the proportions of the cell types in the panel ≤1. In other words, all the cells present in the bulk tissue are (virtually) completely represented by the cell types in the reference panel. When an abundant cell type is missing due to lack of reference data, theoretically, this may lead to errors, as the unrepresented proportion of the bulk tissue will need to be (incorrectly) assigned to an alternative cell type. To explore this, we dropped each cell type in turn from the reference panel, and recalculated the cellular proportion estimates for reconstructed whole blood profiles that included the missing cell type, in increasing proportions. We found that the CETYGO score had a monotonically increasing relationship with the true proportion of the missing cell type (Figure 2). Of note, the magnitude of the CETYGO score in blood data depended on which blood cell type was missing, with the omission of B-cells, leading to the largest errors and the omission of CD8+ T-cells the smallest effect. This is likely due to the methylomic similarity of the two sets of T-cells, whereby CD4+ T-cells are a good alternative to CD8+ T-cells, and suggests that at sites included on the 450K array, B-cells, followed by monocytes have the most distinct profile compared to the average profile of the other cell types. We expanded this framework further to omit up to 3 cell types from the training model, finding that the CETYGO score generally decreases as both the number of cell types in the model increases and the proportion of cells represented in the model increases (Figure 3). However, the distributions of the CETYGO score across different panels of cell types applied to different compositions of whole blood are overlapping and have long tails, highlighting that there are some scenarios where a model with 3 cell types, outperforms a model with 4 or 5 cell types dependent on the abundance of each cell type in the bulk tissue. Exploring the outlier CETYGO scores further, defined as more than 5 standard deviations from the mean (**Supplementary Table 3**), we noted that the worst performing deconvolutions happened when the reference panel included CD4+ T-cells, CD8+ T-cells and NK cells, with up to one other cell type. These three cell types are the most similar in terms of their DNAm profile, and these results suggest that it is challenging to segregate their proportions accurately.

### CETYGO distinguishes nonsense applications

**Figure 4.**
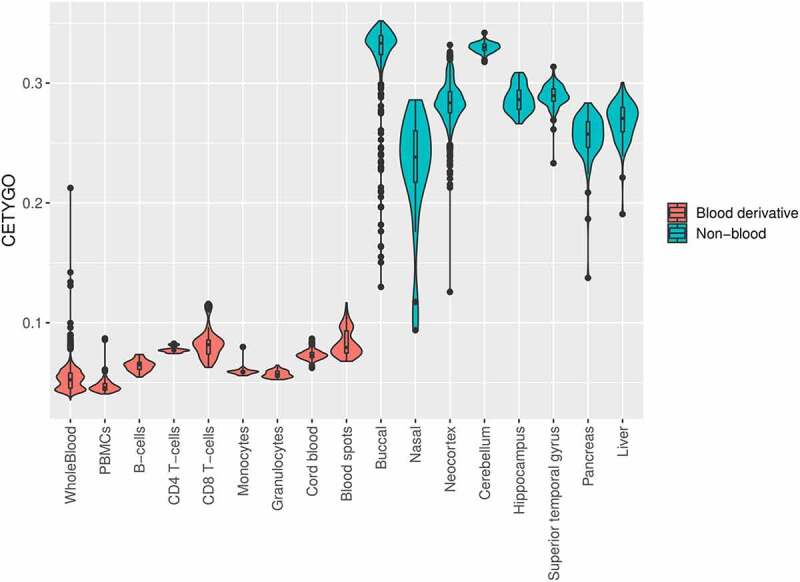
The CETYGO score captures the tissue specificity of deconvolution reference panels. Violin plots of the error associated with estimating the cellular proportions where a reference panel consisting of six blood cell types was applied to 10,447 DNA methylation profiles, across 18 different datasets and 20 different sample types. Each violin plot shows the distribution of the error, measured using the CETYGO score, of the deconvolution (y-axis) grouped by the tissue/cell-type, where the violins are coloured to highlight which samples are derived from blood, which are human derived non-blood bulk tissue, and which are human derived cell-lines.

Having demonstrated the sensitivity of the CETYGO score to detect noisy and incomplete estimates of cellular heterogeneity, we next tested its behaviour when applied to real data in order to provide guidance to the wider research community about how it can be interpreted in the context of epidemiological studies. To this end, we estimated the cellular proportion of six blood cell types and the CETYGO score associated with the estimation for 10,447 DNAm profiles, across 17 different datasets and 17 different sample types (**Supplementary Table 2**). 7,184 (68.8%) of these represent realistic applications as the profiles were derived from blood tissue types and can be used to infer the expected distribution of CETGYO scores across a range of experimental and biological sources. The remaining 3,263 (31.2%) represented ‘nonsense’ applications as these profiles were generated from non-blood samples and can be used to highlight whether the CETYGO score can distinguish sensible deconvolutions. In general, there was a clear dichotomy between the output for these two types of sample; CETYGO scores for blood samples were typically <0.1 and CETYGO scores for non-blood tissues were >0.1 (Figure 4). The median CETYGO score across all whole blood samples was 0.0524 (inter-quartile range = 0.0455–0.0581). Within the whole blood samples there was a bimodal distribution, which on closer inspection appears to be determined by platform, with datasets generated with the 450K array associated with lower CETYGO scores than those generated using the EPIC array (mean difference = −9.11x10^−3^, P = 2.72x10^−223^, **Supplementary Figure 3**). However, it could be that are other technical reasons (e.g. data quality) that underlie this difference. Limiting our comparison to Dataset 8 where we had matched whole blood and purified blood cell types from the same individuals [9], we observed that purified blood cell types were predicted with higher error than whole blood (**Supplementary Figure 4**), with significant differences for all cell types, bar granulocytes (**Supplementary Table 4**). This suggests that it is more challenging accurately to determine when a cell type is pure, than to deconvolute a mixture of cell types. We also noted that the CETYGO score was significantly higher for both cord blood (mean difference = 0.0207; T-test p–value <3.42 × 10^−363^) and neonatal blood spots (mean difference = 0.0307; T-test p–value = 9.19x10^−62^) compared to whole blood. This is in agreement with previous studies suggesting that the standard panel of major blood cell types is not the most appropriate for the assessment of cellular heterogeneity in blood samples obtained for neonatal epigenetic studies [33].

### Cellular heterogeneity estimates are biased by technical factors

**Figure 5.**
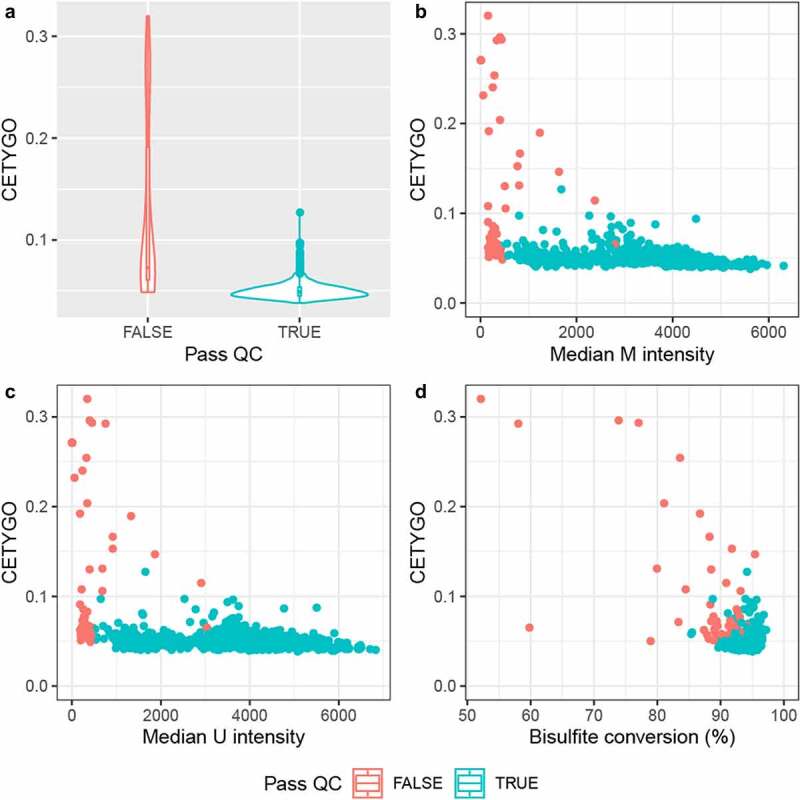
The CETYGO score correlates with metrics of data quality. Summaries of the error associated with estimating the cellular proportions as a function of quantitative metrics of DNA methylation array signal for 725 samples from Dataset 3. **A)** Violin plot of the distribution of the CETYGO score, grouped by whether the sample is of sufficient quality to pass the quality control pipeline. Scatterplots of the error, measured using the CETYGO score (y-axis) for each sample against, **B**) the median methylated (m) intensity across all sites on the microarray, **C**) the median unmethylated (u) intensity across all sites on the microarray, **D**) the bisulpfhite conversion % calculated as the mean across 10 fully methylated control probes. In panels, **B, C** and **D**, the points are coloured by whether the sample passed quality control in panel **A** or not.

While the distribution of CETYGO score across whole blood samples was fairly narrow, we wanted to explore whether CETYGO scores could be used to detect biases in the estimation of cellular composition from whole blood DNAm profiles. In the simulation study we showed that noisy DNAm profiles lead to less accurate estimates of cellular composition. In real data, technically noisy signals should be excluded as part of the pre-processing pipeline in order to improve the power to detect differences between groups. We hypothesized that samples excluded based on technical quality metrics are likely to have higher deconvolution errors as measured by the CETYGO score. Comparing CETYGO scores against standard quality control metrics we found that higher values of the CETYGO score were associated with lower median signal intensities and lower bisulfite conversion statistics (Figure 5), consistent with our hypothesis.

The vast majority of DNAm studies perform normalization to align the distributions across samples, and ultimately make the data more comparable, particularly where data have been generated across multiple batches. We hypothesized that normalizing reference data and test data together to make the genome-wide profiles more similar would attenuate the discriminative signals between cell types and negatively affect the performance of cellular deconvolution. We therefore compared the CETYGO scores calculated with and without normalization of the test data with the reference panel for Dataset 1. In general, the overall distribution of values did not differ dramatically between normalization strategies. However, we did observe that when the reference panel (which is all male) was normalized with the test data, there was a clear bias towards females having higher error (**Supplementary Figure 5**), consistent with analyses showing that normalization can introduce sex effects [34]. In contrast, our adapted method, where we normalized the data separately, was characterized by a dramatically reduced sex difference.

### Cellular heterogeneity estimates are biased by age, sex and smoking status

Across the 6,351 whole blood samples included in our analysis we fitted a linear regression model to test the influence of additional factors on CETYGO scores (**Supplementary Table 5**). As well as the platform effects we described earlier (p-value = 2.72x10^−223^) there were further significant differences between datasets (p-value = 1.75x10^−222^) even after controlling for platform. We also found that every biological factor we tested had a significant association with CETYGO (**Supplementary Figure 6**). This included a negative association with age (coefficient = −7.1x10^−5^, p-value = 0.00215), a positive association with age squared (coefficient = 8.8x10^−7^, p-value = 0.000189), sex (mean difference in males = 9.6x10^−4^, p-value = 4.03x10^−12^) and a positive association with smoking score (coefficient = 6.7x10^−5^, p-value = 1.84x10^−6^).

### Inaccuracies in DNAm prediction algorithms are concordant across predictors for different phenotypes

**Figure 6.**
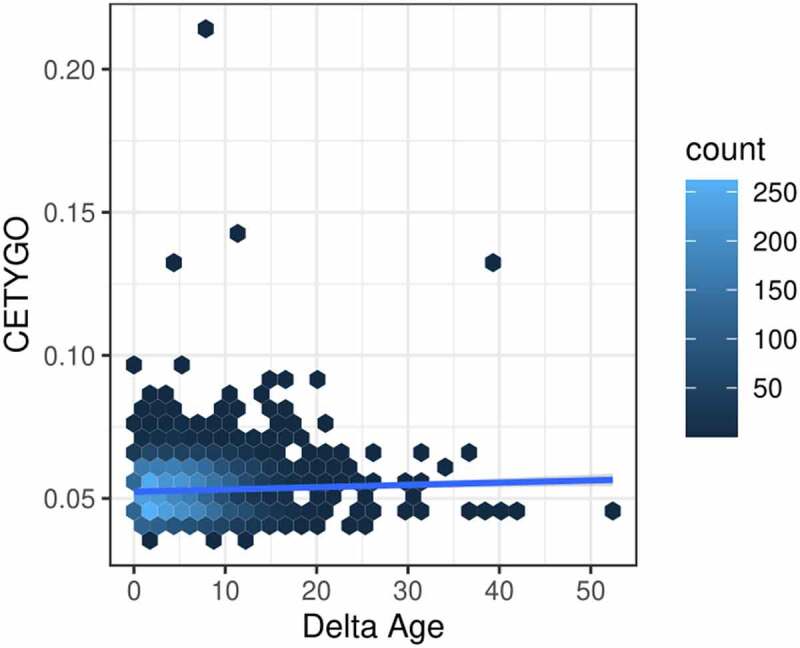
Error in estimation of cellular heterogeneity from DNA methylation data correlates with error from epigenetic clock algorithms. Heatscatterplot of the error measured using the CETYGO score (y-axis), associated with estimating the cellular proportions across 6,351 whole blood profiles against the difference between the sample’s chronological age and age predicted using Horvaths pan-tissue algorithm from the DNA methylation data (delta age; x-axis). The colour of the points represents the density of points at that location.

Finally, we were interested in whether inaccuracy in cellular deconvolution was mirrored by inaccuracies in other epigenetic predictors. Comparing the CETYGO score against the deviation between chronological age and epigenetic age predicted with the Horvath multi-tissue clock [35], we found a nominally significant positive relationship (coefficient = 23.5, p-value = 0.0129) highlighting that samples with inaccurate cellular deconvolution have a larger differences between epigenetic age and chronological age (Figure 6). This suggests that studies which use the residual between epigenetic age and chronological age as a proxy for accelerated ageing may be partly explained by measurement error.

## Discussion

The estimation of cellular composition is vital in epigenetic epidemiology, with these variables being included as co-variates in analyses to minimize the effect of confounding. To compliment these analyses, we have described and validated a novel error metric – CETYGO – that enables the accuracy of the deconvolution to be quantified at an individual sample level. Our results demonstrate that the CETYGO score consistently distinguishes inaccurate and incomplete deconvolutions when applied to reconstructed whole blood profiles and support its inclusion in future DNAm association studies to identify scenarios, or individual cases, when cell composition estimates are unreliable. We have applied it to several existing datasets to further characterize the performance of the predominant application with a reference panel of blood cell types. These analyses provided a number of insights. First, our results indicate that cell types are not equal when it comes to deconvolution accuracy. For example, the omission of B-cells from the standard blood reference panel had the most dramatic effect on their accuracy, while the omission of one of the two types of T-cells had the smallest effect. Furthermore, the model struggled to accurately allocate the abundance of T-cells to the correct subcategory. This is consistent with previous reports that the DNAm profile of B-cells is relatively distinct to that of other blood cell-types, with the profiles of the two classes of T-cells being most similar [9,36]. Second, we highlighted that the estimation of cellular deconvolution using the default 450K reference panel is biased. Specifically, it is less accurate in females, neonates, older individuals and smokers (Figure 4, **Supplementary Table 5**). This has important consequences for epigenome-wide association studies, as it may indicate that existing efforts to adjust for cellular heterogeneity may be less effective in some sets of samples. To minimize this effect, it may be preferable to exclude any sites where either biological (e.g., sex-chromosome linked sites) or technical variation (e.g., cross-hybridizing sites) might be associated with these traits, prior to estimating cellular composition. Previous work has also shown that some of the additional content present on the EPIC array and not present on the older 450K array can be harnessed to improve the accuracy of cellular deconvolution estimation [26]. This would suggest that more recently generated reference panels might be preferable, such as the recent expanded blood panel consisting of 12 leukocyte subtypes [37]. It is unsurprising that the CETYGO scores for neonates were higher, indicative that common blood reference panels derived from adults are not appropriate consistent with previous reported findings [33,36,38]. It is possible this is due to differences in the epigenetic profiles of blood cell types between young and old, or the challenges of extracting DNA from these sample types, leading to increased technical noise. We believe the most pertinent reason, however, is that neonates have blood cells not included in these reference panels, reflecting a situation where an incomplete reference panel was used. Indeed, there are specific reference panels available that include a more appropriate set of cell types for deconvolution of cord blood [33,39,40], and we would hypothesize that the CETYGO score would be lower if these reference panels were used. Altogether, this emphasizes the need to thoroughly benchmark all reference panels and characterize which scenarios they are appropriate for whilst also increasing the diversity of available reference panels.

Our primary motivation was to develop a metric that could be used to assess for an individual sample, how reliable derived estimates of cellular heterogeneity are. To facilitate this we have calculated the CETYGO score in >6,300 whole blood profiles, and provided some guidance about how to interpret the metric. Our data suggest that a CETYGO score >0.1 is consistent with the reference panel not being relevant for the specific tissue being profiled (Figure 4). Although incorrect tissue, had the most dramatic effect, we also found that an elevated CETYGO score can be induced by poor quality DNAm data, where the noise to signal ratio is elevated, generating less sensitive DNAm profiles to the extent that it interferes with the accuracy of the deconvolution model. This can be mitigated by implementing stringent pre-processing pipelines to remove poor quality data. In particular, the principle behind our metric is comparable to the quality control metric DMRSE, which contrasts raw DNAm levels with normalized DNAm levels rationalizing that outlier profiles will require more dramatic transformations to align the data distributions, available in the *wateRmelon* R package [31]. However, even within the pre-processed datasets used in our study there were a handful of samples with outlier CETYGO values. For this reason, we suggest that CETYGO should be added to existing pipelines to provide confidence in analyses that incorporate cellular composition variables. To facilitate this, we have made our method available as a standard alone R package – *CETYGO* – available via GitHub which adapts the existing workflow within *minfi* [32] to simultaneously calculating the CETYGO score alongside the estimation of cellular composition variables using Houseman’s algorithm. In this way it can easily be adapted for use with other available reference panels, both now and in the future. We have also integrated the CETYGO score into the *wateRmelon* function *EstimateCellCounts.wmln()*, used to predict cell type composition, providing users with their deconvolution accuracy estimate when they predict composition.

As well as being able to computationally derive the cellular proportions of the constituent cell-types from a bulk tissue profile, there are now also methods to deconvolute bulk tissue profiles into cell-specific profiles genome-wide [23,41]. These methods are dependent on knowing the cellular proportions of the bulk samples, and if these are derived computationally, we believe it would be prudent to use the CETYGO score to evaluate the accuracy of these prior to deriving the cell-specific profiles. It also plausible that the framework of CETYGO could be adapted to assess the accuracy of the cell-specific DNA methylation profiles. However, given that accuracy is likely to be variable across DNAm sites, it is questionable how valuable a sample-level accuracy score would be in this context, unless it was conditioned on the subset of sites which are predetermined to be associated with highly accurate estimates.

Our findings should be considered in the light of a number of limitations. First, for the purpose of validation, we limited our analyses to the most commonly used deconvolution algorithm, Houseman’s constrained projection approach [17], and the most commonly used bulk tissue, whole blood, for which previously validated reference panels exist [14,25]. Comparisons of the different methodologies for inferring cellular heterogeneity estimates from bulk tissue have concluded that no single method is superior across all test scenarios [20]. Theoretically, though, the concept behind the CETYGO score should be extendable to any reference based deconvolution algorithm or reference panel of cell types and therefore applicable to any tissue, organism, or DNAm profiling technique and could be used to compare the performance of difference algorithms within a single dataset where true cellular heterogeneity is unknown. Second, our method assumes that the cell-specific sites used to estimate cellular composition are not dramatically influenced by any exposure. If differences were induced at these sites, this would cause the error to be overestimated. This assumption is also made by most deconvolution algorithms, and it has been suggested that it is unlikely to be a major concern [42]. Third, we limited the majority of analyses to a reference panel generated with the 450K array and therefore, the conclusions regarding the effect of the specific blood cell types on accuracy may be influenced by the subset of genomic loci included on that technology.

In summary, we have proposed a new metric, CETYGO, to evaluate the accuracy of reference based cellular deconvolution algorithms at an individual sample level. We believe, this tool will be asset in studies of DNAm and have demonstrated how it can be used to assess bias in reference panels, and to identify unreliable estimates of cellular composition.

## Supplementary Material

Supplemental Material
